# The lncRNA TPTEP1 suppresses PI3K/AKT signalling and inhibits ovarian cancer progression by interacting with PTBP1

**DOI:** 10.1111/jcmm.70106

**Published:** 2024-10-18

**Authors:** Yifan Feng, Zhe Zhang, Huijun Yang, Fulu Miao, Yuyang Li, Minmin Zhang, Yunxia Cao, Min Li

**Affiliations:** ^1^ Department of Obstetrics and Gynecology The First Affiliated Hospital of Anhui Medical University Hefei Anhui China; ^2^ NHC Key Laboratory of Study on Abnormal Gametes and Reproductive Tract Anhui Medical University Hefei Anhui China; ^3^ Key Laboratory of Population Health Across Life Cycle Anhui Medical University Ministry of Education of the People's Republic of China Hefei Anhui China; ^4^ Anhui Province Key Laboratory of Reproductive Health and Genetics Hefei Anhui China; ^5^ Anhui Provincial Engineering Research Center of Biopreservation and Artificial Organs Hefei Anhui China

**Keywords:** lncRNA, ovarian cancer, PI3K/AKT, PTBP1

## Abstract

The expression of the long noncoding RNA (lncRNA) TPTE pseudogene 1 (TPTEP1) is significantly downregulated in ovarian cancer (OC). However, the function and mechanism of the lncRNA TPTEP1 in OC have not been identified. To investigate the expression of the lncRNA TPTEP1, we analysed a publicly available dataset and 20 pairs of OC and normal ovarian samples tissue from the First Affiliated Hospital of Anhui Medical University. Functional assays were used to determine the role of the lncRNA TPTEP1 in OC progression. Furthermore, Western blot, FISH, RNA pull‐down, mass spectrometry and RNA immunoprecipitation approaches were used to determine the mechanism by which the lncRNA TPTEP1 affects OC progression. Animal experiments were used to determine the role of the lncRNA TPTEP1 in ovarian tumorigenicity in vivo. The expression of the lncRNA TPTEP1 in OC tissues was significantly lower than that in normal tissues and low expression of the lncRNA TPTEP1 was significantly correlated with advanced FIGO stage and the presence of malignant ascites in OC patients. In vitro and in vivo, regulation of the expression of the lncRNA TPTEP1 caused changes in OC cell proliferation, migration, invasion and apoptosis. Mechanistically, we found that TPTEP1 directly binds to the polypyrimidine tract‐binding protein 1 (PTBP1) protein and inhibits PI3K/AKT signalling. The lncRNA TPTEP1 inhibits PI3K/AKT signalling by directly binding PTBP1, possibly indicating the molecular mechanism underlying its biological function. With further research, these findings may aid in the development of clinically useful strategies for the treatment of OC.

## 1 INTRODUCTION

Ovarian cancer (OC) is the seventh most common cancer worldwide; furthermore, OC has the highestmortality rate among carcinomas of the female reproductive system.^1^ Approximately 70% of patients have advanced OC at the time of diagnosis, and the 5‐year survival rate of patients with advanced OC is only 47.4%.^2^ OC was responsible for 295,414 new cases and 184,799 deaths in 2018.^3^ Therefore, finding therapeutic targets for OC is highly important.

Long noncoding RNAs (lncRNAs) are a class of RNAs that are more than 200 nucleotides in length and have little or no protein‐coding ability.^4^ A number of studies have shown that lncRNAs can participate in various physiological and pathological processes, such as immunity, tumorigenesis and tumour progression, by regulating gene transcription in cis or trans, participating in mRNA processing and regulating the activities of proteins.^5^, ^6^, ^7^, ^8^ An increasing number of studies have shown that lncRNAs can exert their functions by interacting with proteins, nucleic acids and cytokines.^9^ Thus far, accumulating evidence has indicated that lncRNAs are closely related to tumorigenesis, metastasis, prognosis and diagnosis in OC.^10^ For example, the lncRNA RP11‐499E18.1 can play an inhibitory role in OC by regulating the RP11‐499E18.1‐PAK2‐SOX2 axis and lnc00909 functions as a competing endogenous RNA (ceRNA) of MRC2 mRNA by sponging miR‐23‐3p to promote the proliferation, invasion and migration of OC cells.^11^, ^12^

TPTE pseudogene 1 (TPTEP1) is a novel lncRNA. According to previous studies, the lncRNA TPTEP1 can interact with miRNAs or proteins to participate in cell proliferation and migration in many cancers, such as hepatocellular cancer and non‐small cell lung cancer.^13^, ^14^ This finding suggests that the lncRNA TPTEP1 may function as an important regulator in various cancers. Based on its expression level in a dataset from The Cancer Genome Atlas (TCGA), the lncRNA TPTEP1 was identified as one of the significantly downregulated lncRNAs in OC. Hence, the lncRNA TPTEP1 may play an important role in improving the prognosis of OC patients as a potential therapeutic target. However, the function and mechanism of the lncRNA TPTEP1 in OC have not been identified.

In this study, RT–qPCR, Western blotting, RNA pull‐down, mass spectrometry and RNA immunoprecipitation were used to explore the function and mechanism of the long noncoding RNA (long noncoding RNA) TPTEP1 in OC to identify a new therapeutic target for ovarian cancer (OC).

## 2 METHODS

### 2.1 Patient samples, cell lines and nude mice

Fresh tissues were collected from 20 patients with a pathological diagnosis of primary OC who did not receive preoperative adjuvant therapy, and 20 normal ovarian tissue samples were collected from patients with other gynaecological diseases that did not have ovarian involvement but required ovariectomy in the First Affiliated Hospital of Anhui Medical University between 2019 and 2022. After excision, all tissue samples were frozen in liquid nitrogen. Furthermore, we collected the clinical information of OC patients to analyse the relationships between the expression level of TPTEP1 and the clinical characteristics of the OC patients. The study was approved by the Ethics Committee of the First Affiliated Hospital of Anhui Medical University.

The OC cell line SKOV3 used in this study was purchased from Procell Life Science & Technology. OVCA433 cells were a gift from the University of Science and Technology of China. The culture media used for SKOV3 and OVCA433 cells were McCoy's 5a medium and RPMI‐1640, respectively, and both media were supplemented with 10% foetal bovine serum (FBS; Gibco, Australia). The cell lines used in this study were cultured in an incubator with 5% CO_2_ at 37°C, and the culture medium was replaced every 2 days.

A total of eight 6‐week‐old female BALB/c nude mice were purchased from Jiangsu GemPharmatech. All experiments performed with nude mice in this study were approved by the Ethics Committee of the Anhui Medical University. The nude mice were randomly divided into a control group and an experimental group, with four mice in each group. Then, control SKOV3 cells or SKOV3 cells with stable overexpression of the lncRNA TPTEP1 were subcutaneously injected into the left axilla of each control or experimental nude mouse, respectively. After 6 weeks, the nude mice were killed, their tumours were removed and imaged, and the tumour volume was calculated as follows: tumour volume (V, mm^3^) = 0.5 × length × width. Furthermore, each tumour was immersed in formalin and sliced into paraffin sections for immunohistochemical (IHC) staining.

### 2.2 Cell transfection

OC cells were seeded into six‐well plates and Lipofectamine2000 (Thermo Fisher Scientific, USA) was used for in vitro transfection when the cell density confluence 60%. The cells were transfected with a PCDH vector expressing the lncRNA TPTEP1 alone, TPTEP1 and polypyrimidine tract‐binding protein 1 (PTBP1) small interfering RNA (siRNA), or the corresponding control sequences (MiaoLing Plasmid Platform and General Biol, China).

### 2.3 RT–qPCR

Total RNA from tissues and cells was extracted using TRIzol reagent (Thermo Fisher Scientific, USA), and the extracted RNA was reverse‐transcribed into cDNA using a reverse transcription system (Promega, USA). Furthermore, SYBR Green Master Mix (Vazyme, China) was used for quantitative reverse transcription polymerase chain reaction (RT–qPCR) amplification. The expression level of each RNA was calculated using the 2^−ΔΔCt^ method, and the primer sequences used for amplification of the lncRNA TPTEP1, PTBP1 and GAPDH are shown in Table S1.

### 2.4 Western blotting

Total protein was extracted from cells. The proteins were then separated by sodium dodecyl sulfate polyacrylamide gel electrophoresis (SDS–PAGE). The separated proteins were transferred to a polyvinylidene difluoride (PVDF) membrane. The membrane was blocked with 5% skim milk for 1 h at room temperature and then incubated with primary and secondary antibodies. Bound antibodies were detected using enhanced chemiluminescence (ECL). Antibodies against PI3K, P‐PI3K, AKT and P‐AKT were purchased from Cell Signaling Technology (USA); PTBP1, PCNA, MMP2, P53 and BAX were purchased from Abcam (Britain) and Proteintech, Tubulin and GAPDH antibody was purchased from ZSGB‐BIO (China).

### 2.5 5‐ethynyl‐2'‐deoxyuridine (EdU) assay

Cells were seeded on coverslips in a 24‐well plate and cultured at 37°C for 2 h in EdU supplemented medium. The cells were then fixed with 4% formaldehyde for 15 min and permeabilized with 0.3% Triton X‐100 solution. EdU click reaction solution was prepared according to the instructions, and the cells were incubated for half an hour. Finally, Hoechst was used to stain the cells for 10 min, and the cells were observed under an upright fluorescence microscope (100×).

### 2.6 Colony formation assay

Cells were seeded in six‐well plates and incubated at 37°C under 5% CO_2_ for 10–14 days. After that, the cells were fixed with 4% paraformaldehyde and stained with crystal violet solution (Beyotime, China) for 15 min before counting.

### 2.7 Wound healing assay

Cells were seeded in 6‐well plates and incubated in medium supplemented with 10% FBS at 37°C for 24 h. Then, the cell layers on the bottom surface of each well in the 6‐well plates were scratched with a 200 μL pipette tip and washed with PBS 3 times. The cells were cultured in FBS‐free medium and observed under an inverted microscope at 0 h and 24 h after wounding (100×).

### 2.8 Transwell assay

Transwell assays were used to assess the migration and invasion of cells. The membrane in the chamber was coated with Matrigel (Corning, USA) for the invasion assays, and no Matrigel coating was used for the migration assays. The cells were suspended in serum‐free medium, seeded into Transwell inserts and incubated for 24–48 h, fixed with 4% paraformaldehyde and crystal violet solution (Beyotime, China) and counted (100×).

### 2.9 Flow cytometric analysis

Apoptosis of in SKOV3 and OVCA433 cells was assessed using an Annexin V‐APC Apoptosis Detection Kit (Bestbio, China) according to the manufacturer's protocol. In brief, appropriate amounts of cisplatin were added to the experimental and control cells. After 48 h, the cells were resuspended in 500 μL of binding buffer and stained with Annexin V‐APC and PI at 4°C in the dark. Subsequently, apoptosis was assessed by flow cytometry.

### 2.10 RNA sequencing

For lncRNA sequencing, three pairs of cell lines stably transfected with the PCDH TPTEP1 plasmid or the blank plasmid were sent to Genergy for quality evaluation and sequencing. The sequencing data were subjected to differential gene expression analysis and KEGG enrichment analysis, with the R package ggplot2 used for visualization of the results.

### 2.11 Fluorescence in situ hybridization (FISH)

The mixed 5'cy3‐labelled probe was used as instructed with a Fluorescence In Situ Hybridization Kit (GenePharma, China). The in situ hybridization signal was visualized via confocal microscopy (400×). The TPTEP1 probe was designed and synthesized by GenePharma, China.

### 2.12 RNA pull‐down, mass spectrometry and RNA immunoprecipitation (RIP)

A GENERIC biosynthetic biotin‐labelled RNA probe was used for RNA pull‐down with Dynabeads™ M‐280 Streptavidin (Thermo Fisher Scientific, USA). The RIP assay was conducted following the manufacturer's instructions, using a Protein G Immunoprecipitation Kit (Thermo Fisher Scientific, USA). The immunoprecipitated proteins and the bound genes were detected by WB and RT–qPCR, respectively.

### 2.13 Immunohistochemistry

The tumours were removed from the mouse and sectioned. After formalin fixation and paraffin embedding, immunohistochemical analysis was performed using a DAB colourization kit (ZSGB‐BIO, China) following the manufacturer's instructions. An anti‐PCNA antibody (Bioss, China) was used to detect PCNA expression in mouse tumours.

### 2.14 Statistical analysis

The baseline characteristics of the OC patients who were divided into two groups according to the expression level of TPTEP1 are presented as numbers and percentages and were compared via the chi‐square test. The experimental data are expressed as the means and standard deviations, and a *t*‐test was used for comparisons between groups. GraphPad Prism 8.0 (GraphPad Software Inc., San Diego, CA, United States) and R version 4.1.2 (http://www.R‐project ct.org/) were used for all the statistical analyses and graphs generation; * indicates *p* < 0.05, ** indicates *p* < 0.01 and *** indicates *p* < 0.001. *p* < 0.05 was considered to indicate statistical significance.

## 3 RESULTS

### 3.1 The expression of the lncRNA TPTEP1 is downregulated in human OC tissues, and TPTEP1 downregulation is correlated with clinicopathological factors in OC patients

**FIGURE 1:**
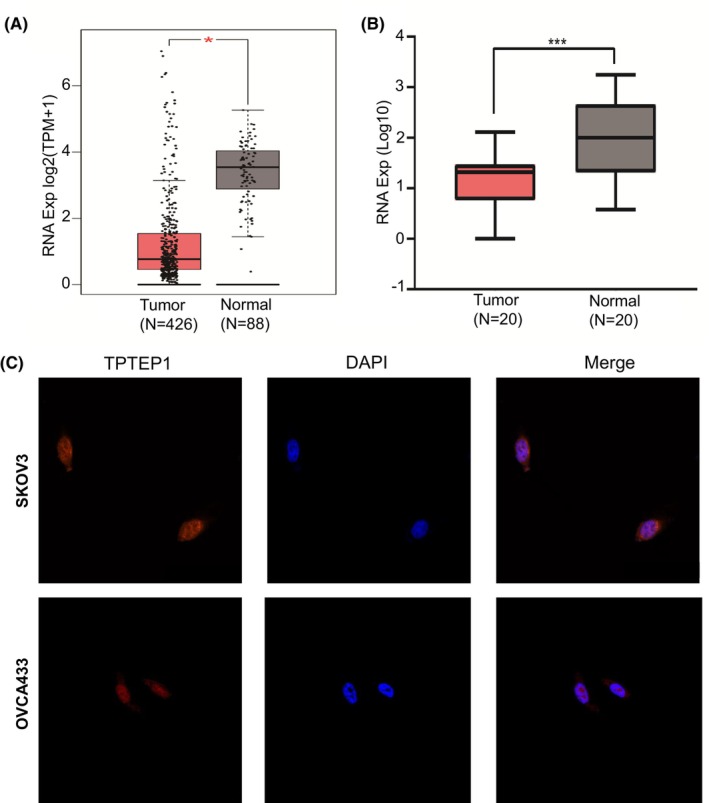
Expression and distribution of the lncRNA TPTEP1 in ovarian cancer. (A‐B) Comparison of the expression of the lncRNA TPTEP1 between ovarian cancer tissues and normal ovarian tissues. (A) GEPIA website (*p < 0.05); (B) First Affiliated Hospital of Anhui Medical University (***p < 0.001); (C) The expression level of the lncRNA TPTEP1 was assessed in SKOV3 and OVCAR433 cells by FISH.

Analysis via the GEPIA server (www.gepia.cancer‐pku.cn) indicated that the lncRNA TPTEP1 was strongly downregulated in 426 OC tissues compared with 88 normal ovarian tissues (Figure 1A). The same pattern was observed in 20 pairs of OC and normal tissues obtained from the First Affiliated Hospital of Anhui Medical University (Figure 1B). Low expression of the lncRNA TPTEP1 was significantly correlated with advanced FIGO stage (*p* = 0.025) and the presence of malignant ascites (*p* = 0.002) in OC patients (shown in Table 1).

**TABLE 1:** The relationship between the expression level of TPTEP1 and the clinical characteristics of OC.

Characteristics	Expression		χ2	*p*
	Low (10)	High (10)		
Age				
<50 years	3 (30.00%)	2 (20.00%)	0.267	0.606
≥50 years	7 (70.00%)	8 (80.00%)		
Differentiation				
Poorly differentiation	7 (70.00%)	7 (70.00%)	<0.001	1.000
Well/Moderate differentiation	3 (30.00%)	3 (30.00%)		
Ascites				
Positive	8 (80.00%)	1 (10.00%)	9.899	0.002
Negative	2 (20.00%)	9 (90.00%)		
FIGO stage				
I/II stage	2 (10.00%)	7 (80.00%)	5.051	0.025
III/IV stage	8 (90.00%)	3 (20.00%)		

### 3.2 TPTEP1 mediates cell proliferation in vitro

**FIGURE 2:**
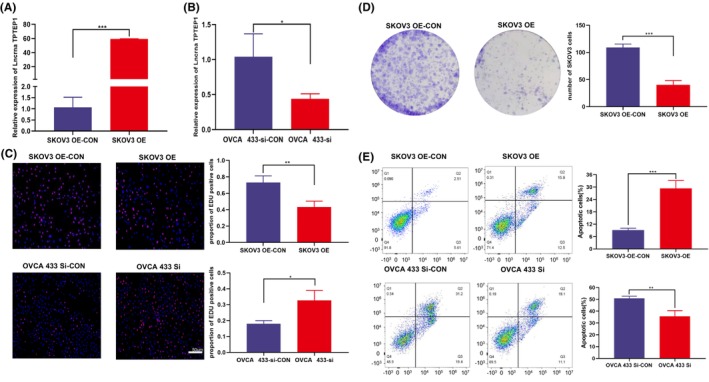
TPTEP1 suppressed OC cell proliferation and promoted apoptosis in vitro. (A‐B) The efficiency of TPTEP1 overexpression (***p < 0.001) and knockdown (*p < 0.05) was detected by RT‒qPCR in the indicated cells transfected with plasmids and siRNAs. (C) EdU assays showing that upregulation of TPTEP1 suppressed cell proliferation in the SKOV3 cell line (**p < 0.01) and that knockdown of TPTEP1 promoted cell proliferation in the OVCA433 cells (*p < 0.05). (D) Colony formation assays for SKOV3 cells after transfection with plasmids (***p < 0.001). (E) Flow cytometry analysis showing that upregulation of TPTEP1 promoted cell apoptosis in the SKOV3 cell line (***p < 0.001) and that knockdown of TPTEP1 suppressed cell apoptosis in the OVCA433 cells (**p < 0.01) .

**FIGURE 3:**
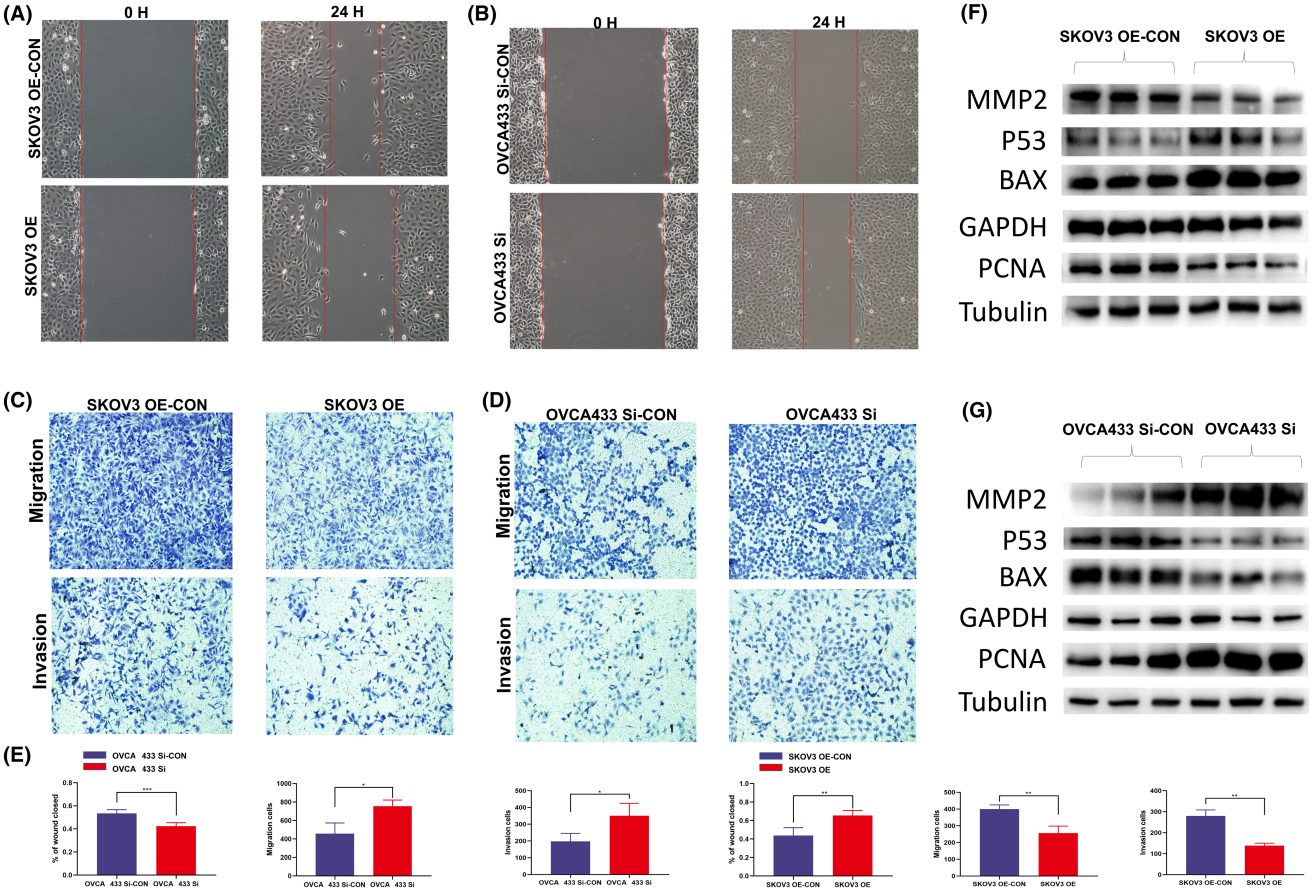
TPTEP1 inhibited OC cell migration, invasion in vitro and WB validation. (A, B) Wound‐healing assay showing that upregulation of TPTEP1 suppressed cell migration in the SKOV3 cell line (**p < 0.01) and that knockdown of TPTEP1 promoted cell migration in the OVCA433 cell line (***p < 0.001). (C, D) Transwell assay showing that upregulation of TPTEP1 suppressed cell migration (**p < 0.01) and invasion (**p < 0.01) in the SKOV3 cell line and that knockdown of TPTEP1 promoted cell migration (*p < 0.05) and invasion (*p < 0.05) in the OVCA433 cell line. (E) Statistics of Wound‐healing and Transwell assay results. (F, G) WB showed the correlation between TPTEP1 expression level and tumour indexes of MMP2, P53, PCNA and BAX.

To evaluate the function of the lncRNA TPTEP1 in OC, we transfected the SKOV3 cell line with the TPTEP1 overexpression plasmid and transfected the OVCA433 cell line with TPTEP1 siRNA. The transfection efficiency was verified by RT–qPCR (Figure 2A,B), and the expression level of the lncRNA TPTEP1 was found to be significantly changed by both overexpression and knockdown. The EdU and colony formation assays revealed that the proliferation of SKOV3 cells overexpressing TPTEP1 was significantly inhibited, while that of OVCA433 cells with TPTEP1 inhibition was promoted, verifying the above results (Figure 2C,D and Figure 3F,G).

### 3.3 Alterations in the expression of the lncRNA TPTEP1 affect OC cell migration, invasion and apoptosis in vitro

To study the effects of the lncRNA TPTEP1 on the invasion and migration of OC cells, wound‐healing and Transwell assays were further conducted. Wounds were introduced into layers of SKOV3‐OE, OVCA433‐si and the corresponding control cells by scratching, and the cells were subsequently cultured for 24 h. Overexpression of the lncRNA TPTEP1 significantly decreased the spreading potential of SKOV3 cells, while downregulation of the lncRNA TPTEP1 significantly increased the spreading potential of OVCA433 cells (Figure 3A,E–G). Additionally, the Transwell assay showed a similar trend with respect to the invasion and migration abilities of SKOV3 and OVCA433 cells (Figure 3C–G). Flow cytometric analysis showed that overexpression of the lncRNA TPTEP1 significantly accelerated apoptosis in SKOV3 cells and that downregulation of the lncRNA TPTEP1 significantly reduced apoptosis in OVCA433 cells treated with cisplatin (Figure 2E–G).

### 3.4 The lncRNA TPTEP1 interacts with PTBP1 and increases its stability

**FIGURE 4:**
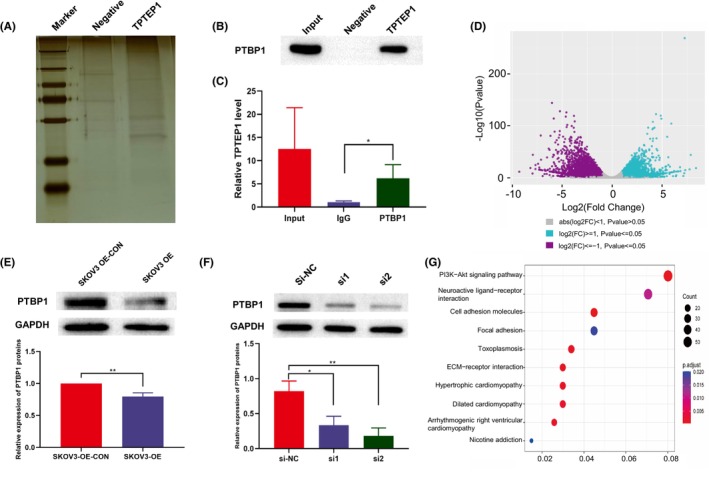
TPTEP1 binds to the PTBP1 protein. (A) RNA pull‐down assay after silver staining; (B) Western blotting to detect PTBP1 protein expression in SKOV3 cells; (C) RIP assay showing that PTBP1 interacted with TPTEP1 in SKOV3 cells (*p < 0.05); (D) The differentially expressed gene of RNA sequencing; (E) PTBP1 protein levels between SKOV3 OE‐CON and SKOV3 OE cells (**p < 0.01); (F) PTBP1 protein levels after transfection of the cells with siRNAs (si1: *p < 0.05; si2: **p < 0.01); (G) The result of KEGG analysis of differentially expressed gene.

According to the above findings, TPTEP1 plays an important role in the progression of OC. Furthermore, according to the FISH results (Figure 1C), the lncRNA TPTEP1 is expressed mainly expressed in the nucleus in SKOV3 and OVCA433 cells; thus, it may interact with proteins to perform its function. Hence, we performed a biotin RNA–protein pull‐down assay to identify the proteins bound to TPTEP1. The bands of interest were analysed by MS, which revealed that PTBP1 might be the target protein of the lncRNA TPTEP1 (Figure 4A,B). Then, the interaction between PTBP1 and the lncRNA TPTEP1 was evaluated by RIP experiments (Figure 4C). Next, we analysed the interaction between the lncRNA TPTEP1 and PTBP1. We found that overexpression of the lncRNA TPTEP1 in SKOV3 cells significantly reduced the protein level of PTBP1 (Figure 4E).

### 3.5 TPTEP1 suppressed OC cell progression, migration and invasion and promoted OC cell apoptosis in a PTBP1‐mediated manner

**FIGURE 5:**
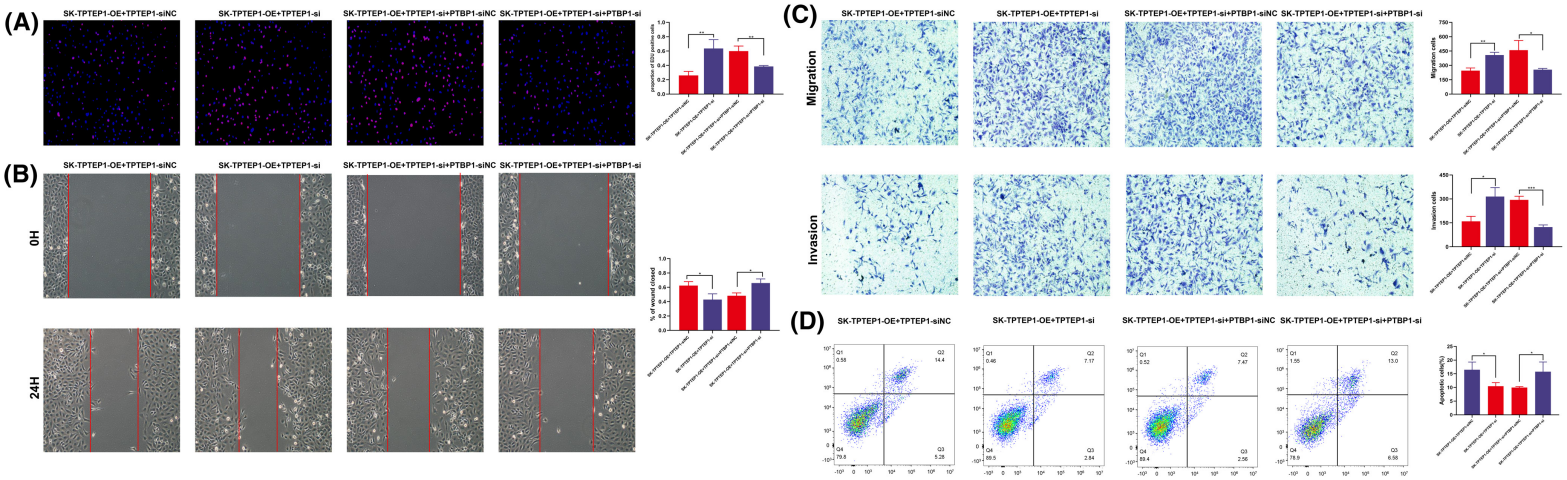
TPTEP1 supressed OC cell progression, migration, invasion and promote OC cell apoptosis in a PTBP1‐mediated manner. (A) EdU assay results showing that knockdown of PTBP1 restore the enhanced cell proliferation by knockdown of TPTEP1 in SKOV3 cells; (B, C) Representative images of the transwell migration and invasion assays and wound healing assays showing that PTBP1 rescued the enhanced invasion and migration abilities by TPTEP1 knockdown. (D) Flow cytometry analysis showing that knockdown of PTBP1 restore the decreased cell apoptosis by knockdown of TPTEP1 in SKOV3 cells.

We next verified whether the lncRNA TPTEP1 plays a role in OC cells by regulating PTBP1. The knockdown efficiency of PTBP1 was verified by Western blotting (Figure 4F). The EdU assay showed that knockdown of the lncRNA TPTEP1 promoted the proliferation of SKOV3‐OE cells, while knockdown of PTBP1 further inhibited cell proliferation (Figure 5A). Similarly, the increases in the invasion and migration abilities and the decrease in the apoptosis rate upon knockdown of the lncRNA TPTEP1 in SKOV3‐OE cells were partially reversed by inhibition of PTBP1 (Figure 5B–D). These findings suggest that the lncRNA TPTEP1 suppresses cell proliferation and metastasis in OC and promotes apoptosis in OC cells in a PTBP1‐mediated manner.

### 3.6 The lncRNA TPTEP1 interacts with PTBP1 to inhibit the PI3K/AKT signalling pathway

**FIGURE 6:**
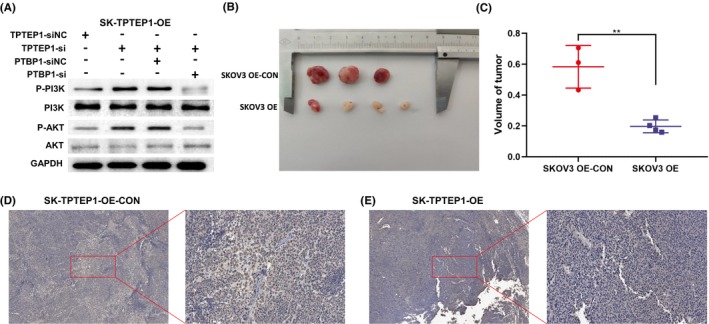
TPTEP1 supressed tumorigenicity in vivo. (A) WB results showing that knockdown of PTBP1 restore the enhanced expression of P‐AKT and P‐PI3K by knockdown of TPTEP1 in SKOV3 cells; (B) Representative images of tumours in nude mice; (C) The nude mouse xenograft model showed that upregulation of TPTEP1 decreased tumour volume compared with control cells (**p < 0.01); (D, E) Representative images of IHC staining for PCNA (5× and 20×).

According to the RNA sequencing results, the lncRNA TPTEP1 was significantly correlated with the PI3K/AKT signalling pathway in OC cells (Figure 4D,G). Previous studies have shown that activation of the PI3K/AKT signalling pathway can promote the progression of OC.^15^ Western blot analysis revealed that downregulation of the lncRNA TPTEP1 increased the levels of proteins in the PI3K/AKT signalling pathway (Figure 6A). In addition, the increases in the levels of proteins in the PI3K/AKT signalling pathway caused by the downregulation of the lncRNA TPTEP1 were significantly inhibited by transfection of si‐PTBP1 (Figure 6A). Overall, our results suggest that the lncRNA TPTEP1 indirectly inhibits the PI3K/AKT signalling pathway by binding to PTBP1.

### 3.7 The lncRNA TPTEP1 mediates tumorigenicity in vivo

To further verify the correlation between the lncRNA TPTEP1 and the proliferation of OC in vivo, SKOV3‐OE‐cells and SKOV3 control cells were injected into the subcutaneous tissues of nude mice. Consistent with the in vitro results, the tumours formed by SKOV3‐OE‐cells were significantly smaller in volume than those formed by control cells (Figure 6B,C). Furthermore, the IHC results showed that the expression of the PCNA protein was significantly increased in the control group (Figure 6D,E).

## 4 DISCUSSION

Ovarian cancer (OC) is the most common gynaecological malignancy. The incidence of OC is expected to increase by 55%, and it is anticipated that the mortality rate of OC will exceed 2.5 million patients per year by 2035 due to increased life expectancy, population growth and economic development.^16^ Hence, revealing the pathogenesis of OC and identifying potential targets for the diagnosis and treatment of OC are highly important for reducing the threat of OC to women's health.

Accumulating evidence indicates that the expression of long noncoding RNAs (lncRNAs) is closely related to the initiation, metastasis or prognosis of cancer.^17^, ^18^, ^19^ By regulating gene expression or central signalling pathways such as the PI3K/AKT pathway, lncRNAs can act as either tumour suppressors or oncogenes.^20^, ^21^ Additionally, recent studies have shown that lncRNAs play an important role in the progression of OC. For instance, the lncRNA HOTAIR has been demonstrated to decrease the invasiveness and tumorigenicity of OC cells by suppressing the expression of TGF‐β1 and ZEB1.^22^ Another study revealed that the lncRNA MALAT1 promotes the malignant progression of OC by regulating epithelial–mesenchymal transition (EMT) through the MALAT1/β‐Catenin axis.^23^ These findings highlight the multifunctional roles and mechanisms of lncRNAs in OC, underscoring the necessity of further exploration in this field.

Previous studies have shown that the expression of the lncRNA TPTE pseudogene 1 (TPTEP1) is widely suppressed in various cancers and that TPTEP1 can inhibit the proliferation, invasion and migration of hepatocellular cancer and non‐small cell lung cancer cells.^13^, ^14^ According to verification experiments with the GEPIA server and tissue specimens, TPTEP1 is also significantly downregulated in OC. Notably, low expression of TPTEP1 was significantly correlated with high‐risk factorsin OC, including the presence of malignant ascites and advanced FIGO stage,^24^ suggesting that the lncRNA TPTEP1 may be closely related to the prognosis of OC patients. Furthermore, the results of EdU, colony formation, wound healing, Transwell, flow cytometry, tumour formation assays in nude mouse, as well as WB analysis, showed that the lncRNA TPTEP1 can inhibit the proliferation, invasion and migration and promote the apoptosis of OC cells.

The interactions between RNAs and proteins are important for cellular processes, including translation, splicing, chromatin modification and translation.^25^ The FISH results revealed that the lncRNA TPTEP1 was expressed mainly in the nucleus, suggesting that it may participate in OC‐related processes by interacting with RNA‐binding proteins. By RNA pull‐down, RNA–protein complexes can be selectively isolated from samples, and information on RNA–protein binding under near physiological can be obtained via RIP assays.^26^ Cross‐validation via RNA pull‐down and RIP assays has been widely used to identify RNA‐binding proteins.^27^

Through the above methods, we found that the lncRNA TPTEP1 regulates the progression of OC by interacting with PTBP1. Polypyrimidine tract‐binding protein 1 (PTBP1) is an RNA‐binding protein that belongs to the heterogeneous nuclear ribonucleoprotein (hnRNP) subfamily and is encoded by a gene located on chromosome 19p13.3 in humans. PTBP1 can regulate the functions of apoptosis, proliferation, migration and invasion via different pathways and molecules in different kinds of cancer.^28^, ^29^ Whether PTBP1 can participate in the progression of OC by binding lncRNAs is worth exploring. The results of our study revealed that the increases in proliferation, invasion and migration and the decrease in the apoptosis rate of OC cells caused by knockdown of the lncRNA TPTEP1 were reversed after knockdown of PTBP1.

PTBP1 has been proven to affect the behaviour of breast cancer and bladder cancer cells or interfere with autophagy in olfactory mucosa mesenchymal stem cells by regulating the PTEN/PI3K/AKT signalling pathway.^30^, ^31^, ^32^ Interestingly, RNA sequencing analysis revealed that the genes differentially expressed between SKOV3 cells stably overexpressing the lncRNA TPTEP1 and the corresponding control cells wexhibited significant enrichment in the PI3K/Akt signalling pathway. In almost all human cancers, including breast cancer and colorectal cancer, the PI3K/AKT signalling pathway is dysregulated, suggesting that the PI3K/AKT signalling pathway plays important roles in tumorigenesis and tumour development.^33^ In OC, the PI3K/AKT signalling pathway is significantly associated with proliferation, platinum‐ resistance and poor prognosis.^15^, ^34^ Our study showed that decreased expression of the lncRNA TPTEP1 led to increased levels of P‐PI3K and P‐AKT, while downregulation of PTBP1 led to decreased expression of P‐PI3K and P‐AKT. Overall, our study indicated that the lncRNA TPTEP1 may regulate the activation of the PI3K/AKT signalling pathway through its binding protein PTBP1, consequently affecting the proliferation, invasion, migration and apoptosis of OC cells.

However, clinical translation of these research findings presents several challenges and necessitates further investigation. To advance towards clinical application, it is essential to validate the prognostic and therapeutic potential of TPTEP1 in larger cohorts and diverse populations. The development of specific lncRNA mimics or inhibitors that can be effectively delivered to tumour sites could significantly improve therapeutic strategies for OC. Recent advancements in nanotechnology and targeted drug delivery systems have shown promise in this area.^35^ In summary, translating the therapeutic potential of TPTEP1 for clinical application requires a multifaceted approach, including validation, targeted drug delivery studies, combination therapy studies and rigorous safety assessments. By addressing these critical issues, we can pave the way for the successful integration of TPTEP1‐based therapies into the clinical management of OC, ultimately improving patient prognosis and quality of life. Additionally, understanding the comprehensive molecular mechanisms of the interaction between TPTEP1 and PTBP1 could identify more targets for precision medicine.

## 5 CONCLUSION

In conclusion, our study confirms the important role of TPTEP1 in ovarian cancer (OC) progression and suggests that TPTEP1 inhibits the PI3K/AKT signalling pathway by directly binding PTBP1, possibly indicating the molecular mechanism underlying its biological function. These findings provide new insights into the role of the lncRNA TPTEP1 in the progression of OC. With further research, these findings may aid in the development of clinically useful strategies for the treatment of OC.

## AUTHOR CONTRIBUTIONS

YFF, YXC and ML performed the study and drafted the article. ML conducted mouse model building, cell culture, data analysis and interpretation. YYL and FLM did IHC western blotting experiment. ZZ and HJY performed the WB. MMZ provides support for language polishing. YXC and ML contributed to the study design. All authors discussed the results and agreed to be accountable for all aspects of the work. All authors read and approved the final manuscript.

## FUNDING INFORMATION

This work was supported by the Natural Science Research Project of Universities in Anhui Province (KJ2019ZD25 and KJ2020A0200).

## Conflict of interest STATEMENT

The authors declare that no competing interests exists.

## Supporting information


**Table S1.**Primer sequence.

## Data Availability

The datasets used and/or analysed during the current study are available from the corresponding author on reasonable request.
