# Supplementary material for: The lncRNA TPTEP1 suppresses PI3K/AKT signalling and inhibits ovarian cancer progression by interacting with PTBP1
Source: J Cell Mol Med. 2024 Oct 18;28(20):e70106. doi: 10.1111/jcmm.70106 (PMC11488117; doi:10.1111/jcmm.70106)
Supplement: Supplementary file 1 — Table S1.Primer sequence. [file JCMM-28-e70106-s001.docx]

| Table S1 | | |
| --- | --- | --- |
| Primer name | Forward (5’-3’) | Reverse (5’-3’) |
| TPTEP1 | CCAGAAAGAAACTCAGCCCAC | TGTGAAGAGACCACCAAACAGG |
| PTBP1 | ATCAGGCCTTCATCGAGATGCACA | TGTCTTGAGCTCCTTGTGGTTGGA |
| GAPDH | GGGAGCCAAAAGGGTCAT | GAGTCCTTCCACGATACCAA |
